# Carpal Tunnel Syndrome in Transthyretin Cardiac Amyloidosis: Implications and Protocol for Diagnosis and Treatment

**DOI:** 10.7759/cureus.14546

**Published:** 2021-04-18

**Authors:** Ryan P Boyle, Josh Sharan, Gary Schwartz

**Affiliations:** 1 Department of Orthopedic Surgery, Nova Southeastern University Dr. Kiran C. Patel College of Osteopathic Medicine, Fort Lauderdale, USA

**Keywords:** carpal tunnel syndome, transthyretin amyloid cardiomyopathy, treatment choices, restrictive cardiomyopathy

## Abstract

Amyloidosis is a group of disorders that occurs due to the aggregation of insoluble and misfolded proteins in the extracellular space, eventually resulting in organ dysfunction. Type II amyloidosis is caused by the deposition of transthyretin (TTR), which will be the main focus of this article. Deposition of TTR in the myocardium results in a restrictive form of cardiomyopathy. TTR can also deposit in the flexor tenosynovium resulting in carpal tunnel syndrome (CTS). CTS develops five to ten years prior to cardiac amyloidosis (CA), and therefore, the temporal relationship allows CTS to be a diagnostic indicator for CA. This report discusses a 65-year-old female and a 76-year-old male, both presenting with pain and paresthesia in the distribution of the median nerve in the left and right wrist. In each case, the diagnosis of bilateral CTS was supported by a positive Phalen’s maneuver and Tinel’s sign. Subsequent tenosynovial and transverse carpal ligament biopsies were performed with Congo red stain revealing amyloid deposits of TTR monomers. This prompted the investigation into possible cardiac involvement. Following cardiac evaluation, the diagnosis of CA was established for the deposition of TTR amyloid monomers. CA has gained much attention in the medical community due to the improvements in cardiac imaging, therapeutic interventions, and diagnostic indicators. Medical professionals should be urged to have a high level of clinical suspicion and refer patients with CTS and select risk factors for cardiac evaluation.

## Introduction

Amyloidosis is a group of disorders that occurs due to the aggregation of insoluble and misfolded proteins in the extracellular space, eventually resulting in organ dysfunction [1]. Amyloidosis can be classified as type 1 or type 2 depending on the protein that is deposited. Type 1 amyloidosis is caused by the deposition of monoclonal immunoglobulin light chains, most commonly the lambda subtype. Type 2 amyloidosis is caused by the deposition of transthyretin (TTR) [1].

Type 2 amyloidosis can be further subclassified into the wild-type TTR amyloidosis (ATTRwt) and the hereditary form of TTR amyloidosis (ATTRm), which results from TTR gene mutations [1]. There have been more than 80 documented TTR mutations, with varying degrees of prevalence and risk of developing symptomatic amyloidosis. Ile68Leu and Glu89Gln, for example, are viewed as cardiogenic mutations [1]. The V30M mutation is the most frequent mutation reported globally, and the TTR Amyloidosis Outcomes Survey found this single mutation to be responsible for more than 70% of symptomatic amyloidosis cases at a single US center [2]. In addition, it has been reported in literature that the Val122Ile mutation affects up to 4% of the African American population [3].

A feared outcome of TTR amyloidosis is cardiac involvement (ATTR-CA), presenting as restrictive cardiomyopathy (RCM). Patients predominantly present with symptoms of right-sided heart failure, as well as lower extremity edema, hepatomegaly, ascites, and elevated jugular venous pressure [2]. Additional organ systems that can be affected include gastrointestinal, renal, hematologic, musculoskeletal, and more.

Musculoskeletal symptoms may manifest as carpal tunnel syndrome (CTS), which is particularly important because of its relationship to systemic and cardiac amyloidosis (CA). The carpal tunnel is located between the carpal bones and the flexor retinaculum and contains the median nerve. The small volume of the tunnel creates a susceptible environment for median nerve compression, obstructing neural transmission. Presenting symptoms typically include numbness, paresthesias, and pain along the median nerve distribution. First reported in 1854, CTS is recognized as the most frequent peripheral neuropathy [4]. CTS has a peak incidence at 40-60 years of age with females twice as likely to be affected than males [5,6]. CTS in the general population has been shown to have a prevalence ranging from 0.2% to 4%, which is drastically increased to 15% to 60% when observing patients with systemic amyloidosis [4,7-11]. The temporal relationship between CTS and CA has also been examined, and it has been determined that CTS precedes cardiac manifestations by 5-10 years [1,2]. Understanding the progression of systemic amyloidosis and the temporal relationship between CTS and CA can be used as a guide for early intervention in an attempt to preserve the myocardium.

## Case presentation

**Figure 1:**
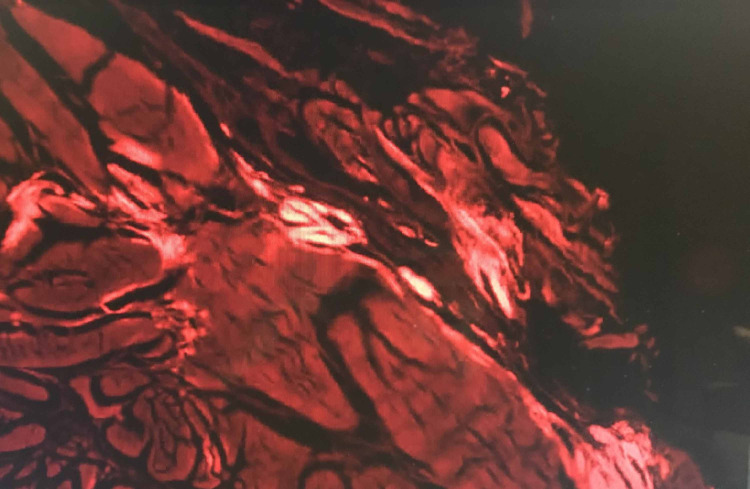
Texas red stain of transverse carpal ligament and tenosynovial fluid.

A 65-year-old female with a significant cardiac history of a past myocardial infarction and hypertension presented with complaints of bilateral pain and numbness in the distribution of the median nerve. She was noted to have a positive Tinel’s sign bilaterally with radiation to the digits and a positive Phalen’s test at 30 seconds. She underwent electromyography (EMG) and nerve conduction studies (NCS), which confirmed the diagnosis of bilateral CTS with active denervation. During the surgical release of the left carpal tunnel, a sample was taken from the flexor tenosynovium and transverse carpal ligament. The samples were studied under Texas red stain and were noted to be positive for amyloid deposition (Figure 1). The sample was subsequently examined via liquid chromatography/mass spectrometry, which revealed a peptide consistent with hereditary ATTR amyloidosis. She was prompted to visit her cardiologist and was subsequently diagnosed with CA.

**Figure 2:**
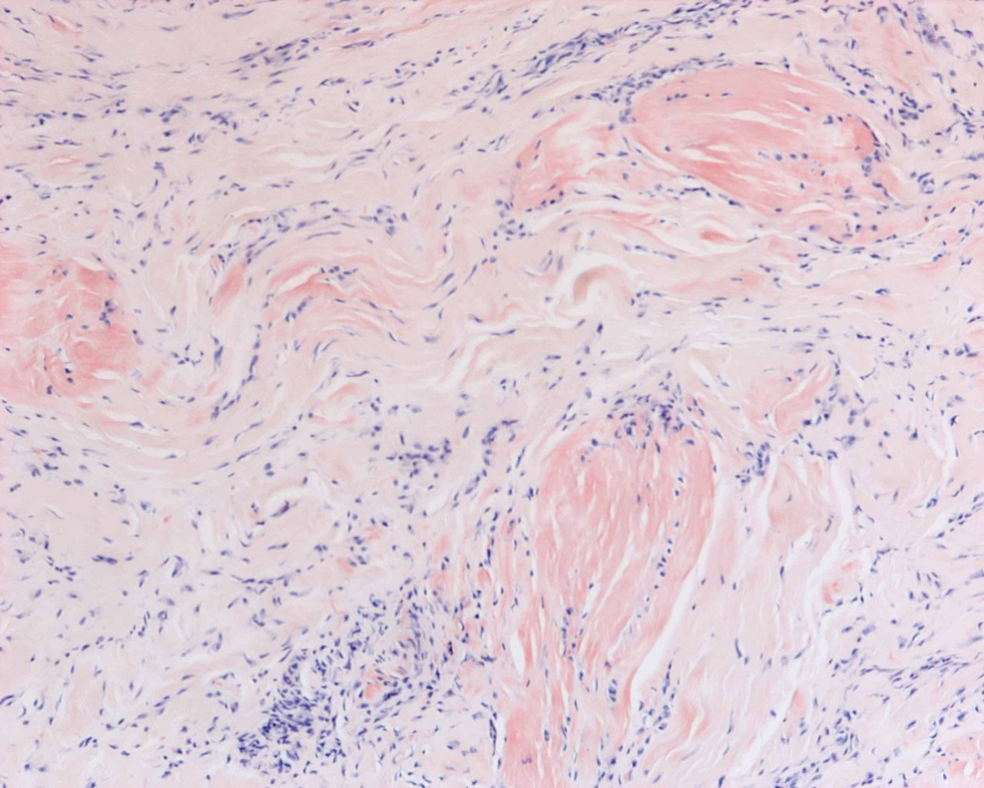
Congo red stain without polarized light of flexor tenosynovium and transverse carpal ligament.

A 76-year-old male with a past medical history of cervical stenosis and hypertension presented with pain and numbness in the distribution of the median nerve at the hands bilaterally. He was noted to have a positive Tinel’s sign at the level of the right carpal tunnel and a positive Phalen’s test at 30 seconds on the right, both of which were negative on the left. The patient underwent EMG and NCS, which revealed bilateral CTS. Samples were taken from the left flexor tenosynovium and transverse carpal ligament and were noted to be positive for amyloid deposition, as can be seen in Figure 2. Light chromatography tandem mass spectrometry was performed and the findings were consistent with age-related TTR amyloidosis. The patient was referred to cardiology and was diagnosed with CA.

## Discussion

In both cases, our patients presented with bilateral pain and numbness in the distribution of the median nerve. Positive Phalen’s test and Tinel’s sign were noted on examinations with radiation to the digits. Patients were subjected to EMG and NCS. Samples were taken during the surgical release of the left carpal tunnel with obtained biopsies and were examined under Congo red stain. Results of each patient showed TTR deposition, confirming the diagnosis of type II amyloidosis. A referral to cardiology was indicated, and upon further evaluation, CA caused by TTR deposition was diagnosed in both patients.

Although CA caused by TTR deposition has a prevalence as low as 1.2% when CTS is absent, prevalence is as high as 5.5% when CTS is present [12]. Furthermore, TTR deposition has been shown to be increased in other soft tissue locations such as the lumbar canal resulting in lumbar spinal stenosis, bicep and quadricep tendon leading to atraumatic tendon rupture, and in the hand with subsequent Dupuytren’s contracture [13].

Diagnosis and treatment

Accounting for approximately 90% of all nerve entrapment pathologies, CTS has a prevalence of 0.2% to 4% in the general population [4,14]. CTS has many etiologies, including pregnancy, rheumatoid arthritis, hypothyroidism, gout, acromegaly, diabetes mellitus, tumors, and in our case, amyloidosis [14]. The clinical presentation with which our patients presented parallels the classic symptoms reported such as numbness, weakness, and paresthesia in the median nerve distribution [14]. In terms of diagnosing CTS, NCS are the gold standard and carry minimal risk [14]. Sensory nerve action potentials (SNAPs) are measured at the mid palm and beyond the carpal tunnel to evaluate possible differences between the two. Studies that show velocities less than 50 meters/second across the tunnel support the diagnosis of CTS [14]. Additionally, a difference in the velocity of the wrist-to-digit segment in comparison to the palm-to-digit segment greater than 10 meters/second adds further support for the diagnosis of CTS [14]. However, in those with normal findings on NCS and a high clinical suspicion for CTS, a combined sensory index test can be performed. This test measures the difference in SNAPs between fingers one and 4 because there is innervation from multiple sources, providing a better statistical comparison [14]. This test has been shown to increase the sensitivity of NCS from 75% to 95% [14]. Furthermore, an EMG can be performed to aid diagnosis by testing for the presence of fibrillation potentials or positive sharp waves in the abductor pollicis brevis muscle [14].

The treatment options for CTS can be classified as non-operative (conservative) or operative. Operative management should be performed after conservative treatment fails to reduce symptoms and improve the quality of life of the patient. Those with mild-to-moderate symptoms can be managed with conservative treatment, which includes splinting, local corticosteroid injections, and physical therapy [15].

After conservative measures have failed, the mainstay management is transverse carpal ligament release [15]. It is strongly recommended to perform an early release of the carpal tunnel to avoid permanent damage to the median nerve as it has been documented that longer duration of symptoms before surgery yields suboptimal recovery [15]. Thus, the outcome of the procedure depends on the duration and preoperative severity of symptoms [15].

**Figure 3:**
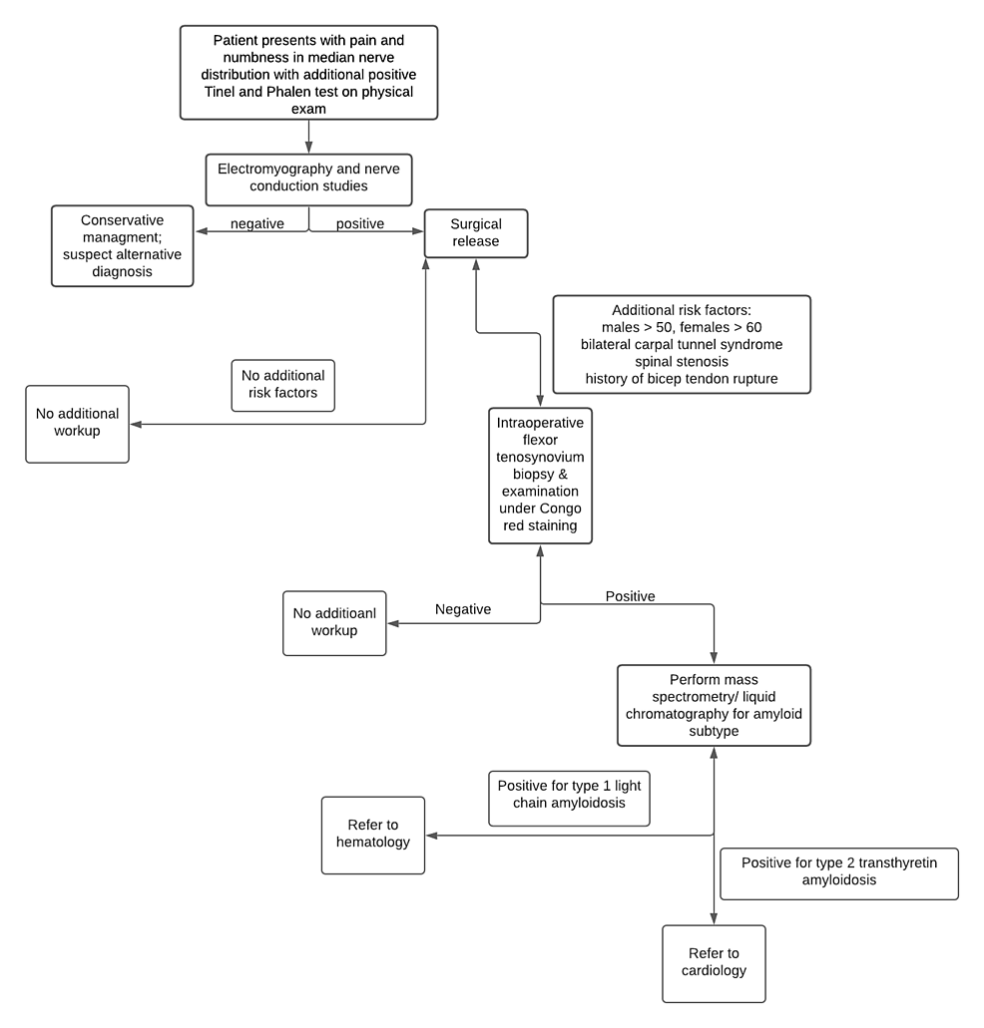
Flow chart of the evaluation of carpal tunnel syndrome and suspected transthyretin cardiac amyloidosis.

CTS without an identifiable cause that fails to improve after conservative measures should raise suspicion for a rarer underlying disease process. Overall, 10.2% of men greater than 50 years of age and women greater than 60 years of age had amyloid identified on tenosynovial biopsy following carpal tunnel release [16]. Of those, 20% showed cardiac involvement after a thorough and comprehensive cardiac evaluation [16]. Although tenosynovial biopsies are not frequently performed, those with significant risk factors could potentially benefit from the procedure to allow for an earlier diagnosis of systemic amyloidosis. After amyloid deposits are detected, mass spectrometry should be performed to avoid false positives from biopsies along with a comprehensive workup for suspected CA [16]. After mass spectrometry has been performed, genotyping of the TTR gene should be done. Extensive evaluation in the workup of ATTR is necessary to avoid misdiagnosis, which occurs relatively frequently [17]. One study showed that 20% of patients without a family history of transthyretin-related familial amyloid-polyneuropathy (TTR-FAP) and 53% of patients with sporadic V30M TTR-FAP were misdiagnosed with chronic inflammatory demyelinating polyneuropathy [17]. Further supporting the frequency of a misdiagnosis, an international survey revealed amyloidosis as being misdiagnosed in 57% of patients with ATTRm and 39% of patients with ATTRwt [16]. Figure 3 shows a flow chart for the evaluation of CTS and ATTR-CA.

CA is the feared outcome of ATTR and a complete workup should be performed to guide early treatment and increase overall survival. Imaging studies are often performed first and show an invariably thickened left ventricular wall, a coexisting pericardial effusion, right ventricular wall thickening, and interatrial septal thickening [3,12]. One study added that clinical suspicion for CA should be strong when left ventricular hypertrophy exists in the absence of hypertension [3].

Amyloid deposits in tissue can be recognized by Congo red or Direct Fast Scarlet 4BS by binding to amyloid and expressing the characteristic apple-green birefringence under polarized light microscopy. This direct visualization of tissue is recognized to be the gold standard for diagnosing ATTR-CA [18]. Due to the different treatment options for light-chain CA and ATTR-CA, endomyocardial biopsies should be performed to confirm the diagnosis and tailor the treatment [18]. Although considered the gold standard, biopsies tend to be invasive and carry an increased risk for infection; therefore, other diagnostic modalities should be considered. One example of an alternative diagnostic technique is positive nuclear imaging with bone-avid tracers [18]. In the absence of monoclonal proteins from type 1 amyloidosis, this method can diagnosis ATTR without histological analysis with high reliability [18]. However, positive results should always be followed up with conventional histopathology with amyloid typing to confirm the diagnosis [16]. Abdominal fat pad biopsies have shown to be unreliable for the diagnosis of ATTR-CA due to their low sensitivity [18]. Additionally, abdominal fat pad biopsies have shown to delay the diagnosis (84% for AL, 15% for ATTRwt, and 45% for ATTR variant) and increase the mortality rate of ATTR-CA patients [18].

A common initial imaging test performed in the workup of ATTR-CA is a transthoracic echocardiogram (TTE). The non-invasive nature of TTE allows it to be more practical compared to the invasiveness of a myocardial biopsy [12]. When performed, thickness of 12 mm or more in the interventricular septum in the absence of alternative causes of left ventricular hypertrophy is a key finding characteristic of ATTR-CA [3,12]. When TTR deposition progresses, a restrictive filling pattern can be seen with a conserved or slightly diminished ejection fraction [12]. In addition to the use of TTE, an electrocardiogram (ECG) can show normal or low ECG voltage often discrepant with TTE findings, along with pseudo-infarcts and poor R wave progression [3]. Predicting the mortality of ATTR-CA can be difficult, and it has been shown that speckle-tracking-imaging-derived global longitudinal early diastolic strain rate was more accurate than conventional diastolic parameters for detecting the mortality rate in those with preserved left ventricular ejection fraction [12].

A newly developed staging system has emerged using B-type natriuretic peptide (NT-proBNP) and effective glomerular filtration rate (eGFR) as variables to determine the overall survival in those with ATTR-CA. The unique parameters for this staging system are the following: Stage 1 is defined as NT-proBNP levels equal to or less than 3000 ng/L and eGFR equal to or greater than 45 mL/minute, stage 3 is defined as NT-proBNP values greater than 3000 ng/L and eGFR less than 45 mL/minute, and stage 2 is defined as those who do not fall into stage 1 or stage 3 [19]. In a review of the data obtained from the study, NT-proBNP levels greater than 3000 ng/L and eGFR less than 45 mL/minute/1.72m^2^ demonstrated a significant correlation with death [19]. The grading system was stratified and the overall survival time for stages 1, 2, and 3 were 69.2 months, 46.7 months, and 24.1 months, respectively [19]. The difference in overall survival times demonstrates the importance of diagnosing CA earlier in its disease course before NT-proBNP levels rise and eGFR declines.

Cardiac magnetic resonance (CMR) imaging is a non-invasive technique that evaluates the structure and function of the heart [12]. A widely used imaging technique for the workup of suspected ATTR-CA, areas of high amyloid deposition show an increased uptake of gadolinium which allows for the visualization of TTR monomers [12]. In those with renal impairment due to systemic ATTR, CMR imaging is useful to evaluate the stroke volume index and global longitudinal and circumferential strength of the heart without utilizing contrast dye [12]. Although many diagnostic values can be obtained with CMR imaging, it may not be readily available and each CMR imaging study is long, thus making CMR imaging a second line in diagnosing ATTR-CA [12].

Radiotracers may be used in conjunction with imaging studies to diagnose ATTR-CA. 3,3- diphosphono-1,2 propanodicarboxylic acid (DPD) uptake can measure the cardiac outcome and visualize myocardial involvement before an echocardiogram can [12]. Different degrees of uptake are measured and range from grade 0-3, with grade 0 showing the best survival rate and grade 3 showing the worst [12]. Tc-PYP tracer uptake with apical sparing of the heart is a known pattern seen in CA caused by TTR [12]. In those with more diffuse ventricular involvement with concomitant apical deposition, patients showed a worse prognosis compared to those who did not [12]. In another study using the Tc-PYP tracer, sensitivity was shown to be as high as 97% with a specificity of 100% [12].

As mentioned earlier, TTR deposits in the ventricles and atria lead to a restrictive form of cardiomyopathy resulting in reduced filling and elevated chamber pressures. This results in symptoms such as pulmonary congestion, dyspnea, and hypotension [3,12]. Treatments for ATTR-CA can be broken down into four categories, including medications to treat the symptoms caused by the restrictive cardiomyopathy, medications that reduce the production of TTR, medications that inhibit the aggregation of amyloid fibrils, and surgical therapies.

Directed treatment towards the restrictive cardiomyopathy sympathology includes loop diuretics and mineralocorticoid antagonists. These two medications alleviate the congestion seen with RCM by decreasing the amount of extra fluid in the intravascular system [18]. Although helpful, the excessive use of loop diuretics may lead to intravascular depletion with subsequent development of prerenal azotemia and hypotension [18]. Thus, it is recommended to add vasopressin V2 receptor antagonists to safely reach euvolemia to avoid these complications [18]. Furthermore, amiodarone, an anti-arrhythmic can be used to treat atrial fibrillation, a frequent manifestation seen with CA. Due to the high frequency of clot formation in the left atrium in those with atrial fibrillation, anticoagulants are highly recommended to avoid complications stemming from the clot [18].

Although liver transplant has been considered a therapeutic option by removing the main source of circulating pathogenic TTR protein, those with the variant form counterintuitively experience increased wild-type TTR deposits following the transplant [18]. These TTR variants act as templates for wild-type TTR production. Thus, combined heart and liver transplant should be considered in patients with advanced disease [18]. According to the International Society for Heart and Lung Transplant guidelines, young patients, for whom age range was undefined in the reference article, should be considered for combined heart and liver transplant (CHLT) to avoid the progression of systemic amyloidosis [18]. Elderly individuals, for whom age range was undefined in the reference article, with wild-type or the Val233Ile variant should be considered for isolated heart transplant [18]. In those who have a definitive diagnosis of ATTR-CA, it is necessary to perform a complete evaluation for CHLT or isolated heart transplantation before disease progression [18].

With advancements in technology and improvements in pharmacological agents, disease-modifying agents have emerged with promising results. The novel pharmacological agent under the generic name of patisiran has shown to significantly decrease NT-proBNP in heart failure patients [18]. Patisiran is a double-stranded small interfering RNA that inhibits the production of both hereditary and wild-type TTR [20]. This drug works by entering the cytoplasm via endocytosis where it targets cellular pathways to decrease TTR production [20]. According to a randomized 18-month trial, the use of patisiran (0.3 mg/kg intravenous once every three weeks) demonstrated a significant improvement in the quality of life of the patient, as well as improved clinical neuropathy scores [18]. Additionally, the drug demonstrated improved cardiac output and global longitudinal strain in ATTR-CA patients [20]. A new trial is being conducted to evaluate patisiran among 300 patients with ATTR-CA. These patients will be randomized to receive 0.3 mg/kg patisiran or placebo over a 24-month period with the aim of determining its efficacy and safety [20].

A Food and Drug Administration (FDA)-approved medication for the treatment of hereditary ATTR-related polyneuropathy is inotersen [20]. Although not approved for the treatment of CA, inotersen has been shown to improve clinical outcomes in patients with ATTR-CA [18,20]. In a randomized 66-week trial, the use of inotersen (300 mg subcutaneous, once weekly) in 172 patients demonstrated improved clinical manifestations, neurological clinical scores, and overall quality of life [18,20]. This agent works by interfering with the production of both variant and wild-type TTR in the liver, thus decreasing the amount of circulating TTR monomers [18,20]. The agent concentrates at high levels in the liver and enters cells via endocytosis. After entering the cell, the drug moves into the nucleus by passive diffusion and active transport interfering with TTR synthesis [20]. In an open-label trial of 15 patients with ATTR-CA, a mean peak reduction in TTR concentration of 72% (range: 39% to 91%) was noted [20]. Side effects seen with inotersen include glomerulonephritis and thrombocytopenia; thus, it is recommended to monitor platelet count weekly and renal function and urinary protein every two weeks [20]. A current study of 50 patients with ATTR-CA is being performed to analyze if the drug can halt the progression of ATTR-CA and determine the tolerability over 24 months [20].

Diflunisal, a non-steroidal anti-inflammatory drug (NSAID) that works by inhibiting cyclooxygenase activity prevents amyloid fibril formation in vitro [18,20]. According to a phase 3 randomized trial, 130 patients diagnosed with ATTR were randomly assigned to 250 mg oral twice daily or placebo for two years, and the results showed a significant reduction in neurologic impairment caused by TTR [18]. Although encouraging findings were noted with neurological symptoms, this agent showed limited beneficial effects on cardiac function [18]. An article reported that it may be considered as an off-label therapy in select patients with ATTR-CA, although more studies are required to determine its potential use in ATTR-CA patients [20]. Due to the risk of renal injury, it is recommended only in those with eGFRs greater than 45 mL/minute/1.73 m^2^ [20]. Furthermore, due to the cyclooxygenase inhibitory mechanism, it is recommended to discontinue other NSAID medications that are being used and to add a proton pump inhibitor to avoid gastrointestinal mucosal injury [20]. Although a low incidence of thrombocytopenia and renal dysfunction has been seen with diflunisal, periodic monitoring of renal status should be considered [20].

Another drug that acts similar to diflunisal by inhibiting TTR aggregation is talcopone [20]. This agent works by inhibiting the catechol-methyltransferase enzyme and binding to the thyroxine-binding pocket at the interface between TTR dimers [20]. This agent is FDA approved for the treatment of Parkinson’s disease; however, its use in patients with ATTR-CA for safety and efficacy is currently being explored [20].

As opposed to preventing TTR formation and aggregation, the combination of doxycycline, an antibiotic, and tauroursodeoxycholic acid (TUDCA), which acts as an antiapoptotic agent, degrades non-fibrillar TTR deposits [18]. In limited trials, it has been reported that the agent attenuated disease progression in ATTR-CA patients [18]. An open-label phase 1/2 trial with 38 patients was conducted using the drug combination of 250 mg of TUDCA three times daily and 100 mg of doxycycline twice daily to determine its tolerability and efficacy in patients with ATTR-CA [18]. Although the trial has been recently completed, results are not reported in this article [18].

As previously mentioned, when TTR tetramers become destabilized, TTR monomers can deposit into various tissue locations causing organ dysfunction. An FDA-approved drug for the treatment of ATTR-CA that can stabilize these TTR tetramers is tafamidis [20]. This agent works by binding to the thyroxine-binding site of the TTR tetramer and increasing the stabilization, thus preventing amyloid deposition [18]. In a phase 3, multicenter randomized trial, patients were randomly assigned to receive 80 mg, 20 mg, or placebo in a 2:1:2 ratio every 24 hours for 30 months [18]. Compared to the placebo, results showed reduced all-cause mortality and cardiovascular-related hospitalizations with tafamidis usage [18}. It was also reported that tafamidis inhibited non-mutant TTR amyloidogenesis in a dose-dependent manner and stabilized the two most clinically relevant mutations of TTR, V30M and V122I [18,20]. Patients on this medication have shown significant improvements in their six-minute walk test and quality of life [20].

It has been recognized in literature that certain mutations such as Val30Met and Val122Ile in the TTR gene can lead to loss of function and result in TTR destabilization with subsequent amyloid deposition [18]. However, a discovered mutation that can lead to a gain of function is the Thr199Met mutation, which results in a hyperstabilized TTR tetramer [20]. This discovery led to the development of a novel drug, AG10, that acts similarly by hyperstabilizing TTR tetramers. The molecular structure of this compound was modified such that optimal binding energetics to the TTR tetramer could be obtained [20]. It was noted that this structure was similar to the motif of the thyroxine-binding site of the Thr119Met mutation, thus allowing for increased stabilization [18]. A randomized, double-blind, phase 2 study of AG10 (400 mg or 800 mg twice daily for 28 days) concluded that AG10 could be safely used in ATTR-CA patients and showed stronger affinity to the TTR tetramer than tafamidis or diflunisal [18,20]. Although confirmed for being well tolerated, a phase 3 prospective trial of AG10 (800 mg for 30 months) has begun to evaluate its safety and efficacy in symptomatic ATTR-CA patients. Results of the study are expected to be released in April 2023 [20].

One compound that has the dual effects of preventing amyloid formation and degrading amyloid fibrils is epigallocatechin-gallate, a polyphenol compound found in green tea. Two observational studies revealed that 12 months of green tea consumption resulted in significant decline in left ventricular mass by 6-13% [18]. However, as these studies are open label and observational with small samples sizes, data are not significant and further research is necessary to determine its efficacy and optimal dosage [18].

## Conclusions

CTS, whether unilateral or bilateral, has shown to be an incremental risk factor for the development of ATTR-CA. This knowledge provides practitioners insight into the workup of possible CA in CTS patients and select risk factors. Although not well established in clinical practice, perioperative biopsies of the tenosynovium and transverse carpal ligament should be obtained during the carpal tunnel release process. This allows an opportunity to detect amyloidosis early in its disease course before CA develops. Proven with the stratified staging system, an earlier diagnosis of systemic amyloidosis with cardiac involvement has shown to lengthen survival times by years. After a specimen has been obtained, Congo red stain of amyloid deposits shows the characteristic apple-green birefringence. Subsequently, mass spectrometry and genotyping should be performed to guide specific treatment. In review of the mentioned pharmacologic treatment options, it is interesting to note that some medications have been approved for the treatment of ATTR-CA, while others are being used as off-label treatment. Although much progress has been made regarding ATTR-CA diagnostics and treatments such as tafamidis showing improved quality of life, many problems are yet to be addressed. Through the collaboration of orthopedic surgeons, general practitioners, cardiologists, and pathologists, establishing a specific protocol with the goal of detecting amyloidosis before CA develops should be prioritized. As we strive closer to this goal, the promotion of CTS as a pre-diagnostic indicator and select risk factor, future growth in research will help providers become more adept at cardiac amyloidosis management.

## References

[1] Milandri A, Farioli A, Gagliardi C (2020). Carpal tunnel syndrome in cardiac amyloidosis: implications for early diagnosis and prognostic role across the spectrum of aetiologies. Eur J Heart Fail.

[2] Gertz MA, Benson MD, Dyck PJ (2015). Diagnosis, prognosis, and therapy of transthyretin amyloidosis. J Am Coll Cardiol.

[3] Mankad AK, Shah KB (2017). Transthyretin cardiac amyloidosis. Curr Cardiol Rep.

[4] Yoshii Y, Zhao C, Amadio PC (2020). Recent advances in ultrasound diagnosis of carpal tunnel syndrome. Diagnostics (Basel).

[5] Farioli A, Curti S, Bonfiglioli R, Baldasseroni A, Spatari G, Mattioli S, Violante FS (2018). Observed differences between males and females in surgically treated carpal tunnel syndrome among non-manual workers: a sensitivity analysis of findings from a large population study. Ann Work Expo Health.

[6] Mattioli S, Baldasseroni A, Curti S (2008). Incidence rates of in-hospital carpal tunnel syndrome in the general population and possible associations with marital status. BMC Public Health.

[7] Rapezzi C, Merlini G, Quarta CC (2009). Systemic cardiac amyloidoses: disease profiles and clinical courses of the 3 main types. Circulation.

[8] Aus dem Siepen F, Hein S, Prestel S (2019). Carpal tunnel syndrome and spinal canal stenosis: harbingers of transthyretin amyloid cardiomyopathy?. Clin Res Cardiol.

[9] González-López E, Gagliardi C, Dominguez F (2017). Clinical characteristics of wild-type transthyretin cardiac amyloidosis: disproving myths. Eur Heart J.

[10] Maurer MS, Hanna M, Grogan M (2016). Genotype and phenotype of transthyretin cardiac amyloidosis: THAOS (Transthyretin Amyloid Outcome Survey). J Am Coll Cardiol.

[11] Rubin J, Alvarez J, Teruya S (2017). Hip and knee arthroplasty are common among patients with transthyretin cardiac amyloidosis, occurring years before cardiac amyloid diagnosis: can we identify affected patients earlier?. Amyloid.

[12] Vidal-Perez R, Vázquez-García R, Barge-Caballero G (2020). Diagnostic and prognostic value of cardiac imaging in amyloidosis. World J Cardiol.

[13] Zegri-Reiriz I, de Haro-Del Moral FJ, Dominguez F (2019). Prevalence of cardiac amyloidosis in patients with carpal tunnel syndrome. J Cardiovasc Transl Res.

[14] Rosario NB, De Jesus O (2020). Electrodiagnostic evaluation of carpal tunnel syndrome. https://www.ncbi.nlm.nih.gov/books/NBK562235/.

[15] Masud M, Rashid M, Malik SA, Ibrahim Khan M, Sarwar SU (2019). Does the duration and severity of symptoms have an impact on relief of symptoms after carpal tunnel release?. J Brachial Plex Peripher Nerve Inj.

[16] Sperry BW, Reyes BA, Ikram A (2018). Tenosynovial and cardiac amyloidosis in patients undergoing carpal tunnel release. J Am Coll Cardiol.

[17] Conceição I, González-Duarte A, Obici L, Schmidt HH, Simoneau D, Ong ML, Amass L (2016). "Red-flag" symptom clusters in transthyretin familial amyloid polyneuropathy. J Peripher Nerv Syst.

[18] Yamamoto H, Yokochi T (2019). Transthyretin cardiac amyloidosis: an update on diagnosis and treatment. ESC Heart Fail.

[19] Gillmore JD, Damy T, Fontana M (2018). A new staging system for cardiac transthyretin amyloidosis. Eur Heart J.

[20] Macedo AVS, Schwartzmann PV, de Gusmão BM, Melo MDT, Coelho-Filho OR (2020). Advances in the treatment of cardiac amyloidosis. Curr Treat Options Oncol.

